# 

**Published:** 1910-09-17

**Authors:** 


### APPOINTMENTS AND RESIGNATIONS.

Dr. Harold Coppock, M.B., Ch.B. (Vict.), has been appointed Assistant Medical Officer to the National Sanatorium, Benenden, Kent.

### Gifts and Bequests.

Mrs. Judith Mellor, of Allerton, Leeds, has left £5,000 to the Oldham Infirmary.

The Treasurers of the Middlesex Hospital have received from Lord Howard de Walden his annual subscription of £300.

The Chelsea Football Club has made a grant of £20 to the Royal Hospital for Incurables, Putney Heath, being part of the proceeds of practice matches.

Mr. Robert Couper Donaldson, of Bournemouth, whose estate was valued at £7,300 net, has left the reversion of his estate to the London Hospital.

Mr. Henry Hawkes, of Leamington, Warwick, has left £105 each to the Warneford Hospital, Leamington, and the Midland Counties Home for Incurables, Leamington, in memory of his late wife.

The Queen has sent from Windsor baskets of black grapes for the patients at the British Hospital for Incurables, Putney Heath, and at the British Home and Hospital for Incurables, Streatham.

The Presbyterian Hospital at New York receives a bequest of nearly £2,000 to found a bed in perpetuity, under the will of the late Mrs. Harriet Coles, of Glen Cove, Long Island.

Mr. Alderman John Heywood, of Bolton, Lancashire, barrister-at-law, J.P. for Lancashire and for Bolton, has left £1,000 to the Bolton Infirmary, £500 to the Bolton District Nursing Association, and £100 to the Manchester Children's Hospital (Bolton Cots Fund).

Mr. Daniel James Woodman, of the Manor Way, Blackheath Park, left estate of the net personalty of £106,490. He bequeathed £1,000 to Guy's Hospital, to found and endow a bed to be called "Woodman's Bed"; £500 each to the Seamen's (*Dreadnought*) Hospital Greenwich, and the Royal Hospital for Incurables, Putney Heath; £300 to the Free Cancer Hospital, Fulham Road; and £200 to the Hospital of St. John the Divine, Lewisham.

The late Mrs. Alice Ann Ryder, of Southport, who left estate valued at £19,055 net, has left, subject to certain provisions for her husband, all of her property to build or endow a hospital or infirmary for the benefit of poor persons of the borough of Middleton, Lancs, in memory of her parents, Robert and Mary Jaques, the recipient of such legacy giving assurances that the monument and grave of her said parents in the cemetery there shall be kept in good order and condition.

### Jottings.

Their Majesties the King and Queen have become patrons of the Hunstanton Convalescent Home and of the Prince Edward Home for Convalescent Children at Hunstanton.

Last week the Countess Beauchamp opened a sale of work at the General Infirmary, Worcester, the object of which was to provide a balcony for the children's Ward where the children can lie in their cots. The work was almost entirely produced by the patients and nurses, past and present. It is hoped that £50 will be realised.

Princess Henry of Battenberg presided last week at the annual meeting of the Frank James Memorial Cottage Hospital, East Cowes, of which she is President. The committee has accepted the offer of Messrs. J. S. White and Co.'s shipbuilding employees to give a complete installation of Röntgen-ray apparatus and to fit up a children's ward and emergency ward for two patients.

As a result of the practice matches of the Leicester Fosse Football Club, the highly satisfactory sum of £107 14s. has been divided by the Club Committee amongst the local charities, the Leicester Infirmary receiving £30, the largest share, and the Leicester Children's Hospital, £10. Cheques have been forwarded to the various societies, and the Club is to be congratulated on the success of the practice matches.

### TO HOSPITAL SECRETARIES AND OTHERS.

We invite contributions from any of our readers, and shall be glad to pay, according to length, for items of news for the institutional section (including appointments, resignations, gifts, etc.) which we may publish. All payments for manuscripts used are made as early as possible after the beginning of each quarter.

